# Supplementary material for: Analysis of PPARγ Signaling Activity in Psoriasis
Source: Int J Mol Sci. 2021 Aug 10;22(16):8603. doi: 10.3390/ijms22168603 (PMC8395241; doi:10.3390/ijms22168603)
Supplement: Supplementary file 1 [file ijms-22-08603-s001.zip › Supplemental materials_Analysis of PPARg signaling activity in psoriasis/Pathway models/Models images and html files/Interleukin-17 and interleukin-22 signaling in psoriasis/1007279293.html]

PI3K ->-> PIP3


# ChemicalReaction PI3K ->-> PIP3

|  |  |
| --- | --- |
| URN | urn:agi-chemicalreaction:in:urn:agi-aopfc:0000447:out:urn:agi-cas:165689-82-7 |
| Connectivity | 2 |
| References | 252 |
| Effect | unknown |
| ObjectType | ChemicalReaction |
| RelationNumberOfReferences | 241 |
| RelationConfidenceLevel | 3 |
| Original # of References | 277 |

---
